# Determinants of Early Neonatal Weight Loss in Historical Swiss Maternity Hospital Records

**DOI:** 10.1002/ajhb.70349

**Published:** 2026-10-20

**Authors:** Anton Nagaroor, Mathilde Le Vu, Katarina L. Matthes, Kaspar Staub

**Affiliations:** ^1^ Institute of Evolutionary Medicine, University of Zurich Zurich Switzerland; ^2^ Univ Rennes, Inserm, EHESP, Irset (Institut de Recherche en Santé, Environnement et Travail), UMR_S 1085 Rennes France

**Keywords:** developmental plasticity, historical epidemiology, infant feeding, neonatal adaptation, neonatal weight loss

## Abstract

**Objective:**

Early neonatal weight loss is an aspect of neonatal adaptation influenced by maternal, nutritional, and perinatal factors. Determinants of early neonatal weight loss in historical populations remain poorly understood. This study investigated determinants of neonatal weight loss as an early postnatal adaptation phenotype using Swiss maternity hospital records from 1905 to 1924, including the influence of the First World War and the 1918–1920 influenza pandemic.

**Methods:**

Birth records from Lausanne Maternity Hospital (1905–1924) were analyzed. Maximum percentage neonatal weight loss was calculated during hospitalization, excluding implausible trajectories (> 20% loss) and weight gains. Associations with feeding type, historical exposures, and maternal health were assessed using multivariable linear and logistic regression for maximum and high (> 10%) weight loss, respectively. Stratified and sensitivity analyses assessed effect heterogeneity and model robustness.

**Results:**

The analytical sample included 11 739 singleton live births. Mean maximum observed neonatal weight loss was 6.7% (SD 3.5%), and 16.8% experienced high weight loss. Compared with artificial feeding, breastfeeding was associated with lower maximum weight loss (β = −1.07 percentage points, 95% CI −1.32 to −0.81) and lower odds of high weight loss (OR = 0.56, 95% CI 0.47–0.68). Mixed feeding was associated with greater weight loss and higher odds of high weight loss. Pandemic exposure was associated with slightly greater weight loss, whereas the association with First World War exposure was small and did not provide clear evidence of an independent association.

**Conclusion:**

Feeding mode was the strongest determinant of early neonatal weight loss. Breastfeeding was consistently associated with lower weight loss, while pandemic exposure and maternal influenza during pregnancy were associated with slightly higher weight loss. These findings suggest that neonatal weight loss may provide an early postnatal indicator of neonatal adaptation reflecting both immediate nutritional influences and broader prenatal conditions.

## 1 Introduction

Early neonatal weight loss is a typical physiological phenomenon after birth and during the first days of life and is regained during the first 10–14 days of life (Eren and Eren 2026). Newborns lose weight due to fluid shifts, meconium passage, and the transition from placental nutrition to enteral feeding (Macdonald et al. 2003). From a human‐biological perspective, this transition also marks the shift from prenatal maternal provisioning to postnatal nutritional dependence, with early weight trajectories potentially reflecting both infant physiological adaptation and the establishment of postnatal feeding. In healthy term infants, this early weight loss is generally considered physiological; however, high neonatal weight loss may indicate inadequate fluid or milk intake, breastfeeding difficulties, dehydration, jaundice, or other neonatal complications (Dewey 2001). Therefore, neonatal weight loss can serve as an early clinical indicator of both neonatal adaptation and maternal and infant health, particularly in the context of feeding and postnatal care (DiTomasso and Cloud 2019; Miyoshi et al. 2020). Several maternal, neonatal, and obstetric factors influence the extent of neonatal weight loss. Previous studies identified feeding mode, delayed lactogenesis, breastfeeding difficulties, maternal body mass index, parity, gestational age, birthweight, mode of delivery, neonatal sex, and maternal intrapartum fluid administration as important determinants of postnatal weight trajectories (Miyoshi et al. 2020; Regnault et al. 2011). A systematic review of neonatal weight loss among breastfed newborns showed that neonatal weight loss is greatest after the third day following birth and is regained within 10–14 days, which represents a common component of normal neonatal adaptation (DiTomasso and Cloud 2019).

However, most previous studies were conducted in modern neonatal settings with contemporary feeding practices, medical care, and nutritional conditions (DiTomasso and Cloud 2019). Less is known about neonatal weight trajectories in early 20th century populations exposed to nutritional or war‐related crises, infectious disease burden (including during pandemics), socio‐economic insecurity, and limited obstetric interventions. Such crisis‐related exposures may plausibly have influenced postnatal adaptation through impaired maternal nutrition, altered breastfeeding capacity, maternal illness, and increased neonatal vulnerability (Beach et al. 2022). These pathways can also be considered within the concept of maternal capital, which describes the phenotypic resources available to mothers for investment in their offspring (Wells 2010, 2025). Maternal resources may influence offspring development during pregnancy and lactation and may themselves be constrained by nutritional, infectious, and broader societal adversity (Wells 2010, 2025). Maternal capital may provide offspring not only with resources but also with buffering against short‐term ecological fluctuations. Through maternal physiology, external environmental variation may be smoothed during sensitive periods of development; however, when an environmental stressor becomes embodied in maternal physiology, this buffering may be reduced, and the fetus may be more directly exposed (Wells 2003).

Historical maternity records provide a unique opportunity to study neonatal health under conditions that differed substantially from modern obstetric settings. Such data often contain detailed information on maternal characteristics, pregnancy, birth, neonatal/maternal anthropometry, feeding practices, and postnatal development, allowing reconstruction of early‐life health trajectories in populations exposed to infectious diseases, nutritional deprivation, and limited obstetric interventions (Beach et al. 2022; Miyoshi et al. 2020). Historical epidemiological analyses may therefore improve the understanding of vulnerability and developmental adaptation processes that remain relevant today, as early‐life exposures can have lasting consequences for health and development (Barker 1990; Roseboom et al. 2001). Importantly, such records also allow early postnatal outcomes to be examined at the intersection of prenatal developmental conditions, maternal characteristics, and the postnatal nutritional environment.

The period surrounding the First World War and the 1918–1920 influenza pandemic (Spanish Flu) represents a particularly informative historical setting to examine a fundamental and profound crisis situation (Beach et al. 2022; Miyoshi et al. 2020). Although Switzerland remained neutral during the First World War, the population nevertheless experienced strong food shortages, noticeable malnutrition, economic hardship, and broader societal disruption, in particular towards the end of the war (Krämer et al. 2016). At the same time, the 1918–1920 influenza pandemic represented a major public health crisis. More than half of the Swiss population is estimated to have been infected, including many pregnant women, and approximately 25 000 deaths were recorded. Mortality was particularly high among young adults. The pandemic is widely regarded as the greatest demographic catastrophe in Switzerland during the 20th century (Sonderegger and Tscherrig 2016; Ziegler et al. 2025). Together, these concurrent exposures created a unique historical setting in which pregnant women and newborns were exposed to multiple biological and environmental stressors. Studying neonatal adaptation in this context may therefore provide insights into how maternal illness, nutritional adversity, and crisis‐related exposures influence early‐life health, with potential relevance for contemporary populations affected by pandemics, malnutrition, or humanitarian crises (Floris and Staub 2025; Roseboom et al. 2001; Scholte et al. 2015). In this area, historical studies on early 20th century maternity hospital data from Basel, Bern, Helsinki, Barcelona and other cities have already demonstrated that maternal undernutrition and infectious diseases during pregnancy negatively affected neonatal outcomes such as birthweight, placenta weight, head circumference, or mortality indicators (Butie et al. 2020; Floris and Staub 2025; Floris et al. 2016; Galofré‐Vilà and Harris 2021; Saaritsa 2026; Salvisberg et al. 2023).

Prenatal adverse exposures, including maternal infections and nutritional deprivation, are known to influence fetal growth and birth outcomes. Within developmental plasticity frameworks, such exposures may affect fetal growth and physiological development through changes in the maternal–placental–fetal environment, with potential consequences extending into the neonatal period (Wells 2025). Evidence from both contemporary and historical populations has linked maternal infections, including malaria, congenital cytomegalovirus infection, syphilis, and influenza, as well as severe maternal undernutrition, to impaired fetal growth, lower birthweight, smaller neonatal anthropometric measures, and adverse perinatal outcomes (Bale et al. 2002; Dombrowski et al. 2019; Stein and Susser 1975). Consistent with this broader literature, recently collected data from the maternity hospital in Lausanne demonstrated that maternal influenza‐like illness during the 1918 influenza pandemic was associated with lower birthweight, smaller head circumference, lower placental weight, and increased risks of preterm birth and low birthweight (Le Vu et al. 2025). In the same study population, maternal syphilis was also associated with impaired neonatal anthropometric outcomes (Le Vu et al. 2024), while maternal–fetal body size mismatch and indicators of poor maternal nutritional status were linked to more complicated deliveries and adverse obstetric outcomes (Haag et al. 2025). However, no study has yet examined early neonatal weight loss within this population and historical time period.

Against this background, we conceptualized early neonatal weight loss as a short‐term postnatal adaptation phenotype potentially influenced by both prenatal developmental conditions and the postnatal nutritional environment. The aim of this study was to identify determinants of early neonatal weight loss using the Lausanne dataset. We included feeding mode, exposure to periods of crisis (the First World War and the 1918–1920 influenza pandemic), and other maternal and neonatal factors as potential factors affecting neonatal weight loss. Since we expect neonatal weight loss to be particularly dependent on feeding mode, we further tested whether its effect was modified among clinically (low birth weight, preterm, exposure to maternal flu, neonatal sex) or contextually (pregnancy during periods of crises) vulnerable neonates.

## 2 Materials and Methods

### 2.1 Data Source and Study Population

This study reused historical birth records from the Swiss maternity hospital in Lausanne collected and recorded during the early twentieth century, a period characterized by substantial social, nutritional, and epidemiological changes, including the years surrounding World War I and the 1918–1920 influenza pandemic. Similar studies based on other historical maternal hospital datasets in Switzerland and beyond demonstrated associations between maternal health, crisis‐related exposures, and neonatal anthropometric outcomes during the first half of the 20th century (Butie et al. 2020; Galofré‐Vilà and Harris 2021; Miyoshi et al. 2020; Salvisberg et al. 2023).

The present study reuses digitized archival birth records originating from the maternity hospital of Lausanne (Haag et al. 2025; K VIII e 187‐566 Maternité/Obstétrique/Gynécologie, 1898–1948, 1898; Le Vu et al. 2024, 2025). The hospital systematically documented births and collected detailed medical, demographic, anthropometric, and obstetric information on mothers and newborns since the end of the 19th century (Haag et al. 2025; Le Vu et al. 2025). The archived birth registers included women from different socioeconomic backgrounds and documented both uncomplicated and complicated births. Individual‐level information was transcribed from historical maternity hospital records preserved in the cantonal archives. Each birth entry contained detailed information on maternal characteristics, pregnancy history, delivery conditions, neonatal anthropometric measurements, feeding practices, and repeated postnatal weight measurements obtained during the postpartum period. Detailed information about these archival records and how variables were transcribed can be found in previous studies (Haag et al. 2025; Le Vu et al. 2024, 2025).

For the present study, singleton live births with available birthweight data and at least one postnatal weight measurement during the first days of life were included. Miscarriages, stillbirths, and cases with missing key anthropometric information were excluded from the study sample; details are provided in Section 3.

### 2.2 Variable Definitions

#### 2.2.1 Neonatal Weight Loss

The primary outcome of this study was early neonatal weight loss during the first days of life. Postnatal weight measurements were transcribed for day 5 and 10 after birth; if these two measures were not available, the closest measurement was transcribed. The largest observed percentage decrease among the available postnatal measurements was defined as the primary outcome variable and used as an approximation of maximal early neonatal weight loss. Because measurements were primarily available around days 5 and 10, this measure represents an approximation of the true postnatal weight nadir. For each newborn, percentage weight loss was calculated separately for both postnatal measurements using the following formula:

$$\text{Weight loss}\;\left(\%\right)=\frac{\left(\text{Birthweight}-\text{Postnatal weight}\right)}{\text{Birthweight}\;}\times 100$$

Expressing neonatal weight loss relative to birthweight allows standardized comparison across newborns with different birthweights and gestational ages (Butie et al. 2020). The days of the first and second postnatal weight assessments and the measurement day corresponding to maximal observed weight loss were also assessed. Physiological neonatal weight loss during the first postnatal days primarily reflects postnatal fluid adaptation and transitional feeding processes. Weight loss exceeding approximately 10% of birthweight is generally considered clinically relevant (National Guideline Alliance (UK) 2017; World Health Organization 2015).

Based on biological plausibility and literature indicating that physiological neonatal weight loss rarely exceeds 10%–15% of birthweight, analyses were restricted to neonatal weight loss values below 20%. Observations outside this range were considered implausible and likely reflected measurement errors in the historical records.

Because the objective of this study was to characterize early neonatal weight loss, observations showing weight gain during the early postnatal period were considered unlikely to represent the physiological weight‐loss phase (Macdonald et al. 2003) and were therefore excluded. Such observations most likely reflected weight measurements obtained after recovery of birthweight or documentation inconsistencies within the historical records. Overall, 397 observations (3.27%) were excluded because the recorded postnatal weight trajectories were inconsistent with the study objective of assessing early neonatal weight loss. These observations either showed weight loss (> 20% of birthweight) (*n* = 88, 0.73%) or weight gain (*n* = 309, 2.55%) during the early postnatal period. Although only 3.27% of observations were excluded based on predefined biological plausibility criteria, excluded infants had somewhat less favorable neonatal characteristics than included infants. Therefore, a small degree of selection bias due to underrepresentation of clinically vulnerable newborns cannot be excluded.

#### 2.2.2 Feeding Categories

During the early 20th century, artificial feeding differed substantially from modern infant formula feeding. Commercial infant formulas were not yet widely available, and artificial feeding typically consisted of animal milk (most commonly cow's milk), diluted milk preparations, or other milk substitutes prepared according to contemporary infant‐feeding practices (Mepham 1993; Vögele et al. 2013). Consequently, the historical feeding categories should not be interpreted as directly comparable with modern formula‐feeding practices.

The original feeding variable in the sources contained several detailed historical textual categories, including maternal feeding, artificial feeding, mixed feeding, and sequential changes between feeding types during hospitalization. To improve interpretability and avoid unstable estimates due to small category sizes, feeding information was recoded into broader categories. Newborns recorded in the source as receiving “maternal” milk were classified as breastfed. Categories indicating both maternal and non‐maternal feeding were grouped as mixed feeding, whereas artificial feeding and maternal‐to‐artificial feeding were classified as artificial feeding. The historical category “maternal‐to‐artificial feeding” indicated a transition from maternal milk feeding to artificial feeding during the hospital stay. Rare or heterogeneous feeding categories, including records labeled as “human milk” and miscellaneous feeding descriptions, were grouped into an “other” category because of limited case numbers and uncertainty regarding the precise historical feeding practices represented by these records. The “other” category remained included in regression analyses but was interpreted cautiously because of its heterogeneity.

#### 2.2.3 Gestational Age and Birthweight

Gestational age was assessed as categorical information at birth by the physician and could also be calculated based on the self‐reported date of last menstruation and the date of delivery recorded in the source. If the date of last menstruation was missing, or if physician‐assessed and calculated gestational age were discordant, gestational age as assessed by the physician at birth was used (Le Vu et al. 2024). Preterm birth was defined as gestational age < 37 completed weeks. Birthweight was analyzed both continuously and categorically, with low birth weight defined as birthweight < 2500 g.

#### 2.2.4 Other Neonatal and Maternal Variables

The historical birth records also contained information on the mother's age (in years, as a continuous variable), the child's sex, and the parity of the birth. Neonatal mortality was defined as a documented postnatal death occurring within the first 28 days of life. In addition, the sources also contained a wide range of qualitative information on the mothers' health status. Such information included assessments by the physician on maternal nutritional status (obesity, underweight), on the presence of a goiter, on maternal infections, and other clinical observations.

A categorical variable called “General health status” was created based on this qualitative information, coded from 1 to 3, with higher numbers indicating poorer maternal health (Le Vu et al. 2025). For example, if there were none of these indications (obese, thin, goiter, rickets, infection), health status was = 1 (good). If there was albuminuria, eclampsia, or other very severe/life threatening conditions being mentioned in this section of the records, it was = 3 (very bad). Everything in between was coded as 2 (bad health status).

#### 2.2.5 Crisis Exposure Variables

Pregnancy‐related exposure to First World War and the influenza pandemic was determined using estimated conception dates derived from gestational age information (Butie et al. 2020; Le Vu et al. 2025). Pregnancies were classified as exposed if any part of gestation overlapped with the First World War (July 1914–November 1918) or with the major influenza pandemic waves (July 1918–March 1919 and January 1920–March 1920). Information on maternal influenza‐like illness during pregnancy was obtained from physician‐recorded diagnoses documented in the original maternity records and was analyzed as an additional exposure variable, as described in previous studies using the same historical records (Le Vu et al. 2024, 2025).

### 2.3 Statistical Analysis

Descriptive analyses were performed to summarize maternal, neonatal, feeding‐related, and obstetric characteristics of the study population. Continuous variables were described using means and standard deviations or medians and interquartile ranges, depending on data distribution. Categorical variables were summarized using absolute and relative frequencies.

The primary outcome was maximum observed neonatal weight loss (%) during the early postnatal period. Associations between neonatal, maternal, and feeding‐related factors and maximum observed neonatal weight loss were evaluated using multivariable linear regression models. Main models were adjusted for gestational age, because birthweight is part of the calculation of percentage neonatal weight loss and was therefore not included as an adjustment variable in the primary models. Gestational age is a major determinant of birthweight, and adjustment for both variables may lead to overadjustment or collider‐stratification bias, particularly when estimating associations with neonatal outcomes (Wilcox et al. 2011). Therefore, separate model specifications were used: one adjusted for gestational age (main analyses) and one adjusted for birthweight (sensitivity analysis). This approach allowed assessment of the robustness of the findings without conditioning simultaneously on two causally linked birth characteristics. All models included feeding category, historical exposure variables, neonatal sex, and continuous parity and maternal age as covariates. The secondary outcome was high neonatal weight loss as a binary variable, defined as weight loss exceeding 10% of birthweight. Associations with high neonatal weight loss were examined using multivariable logistic regression models. As for the linear analyses, gestational‐age‐adjusted models were considered the primary analyses, whereas birthweight‐adjusted models were conducted as sensitivity analyses.

To evaluate whether historical crisis exposure influenced neonatal weight loss, pregnancy‐based exposure variables were included in the main multivariable regression models. Pregnancies were classified according to whether gestation overlapped with the First World War and/or the influenza pandemic. These models examined whether neonatal weight loss differed according to maternal exposure to historical crises during pregnancy while accounting for feeding type and other covariates. To assess potential confounding by indication, whereby alternative feeding regimens may have been preferentially administered to clinically vulnerable mothers or newborns, maternal and neonatal characteristics were compared across feeding categories. Continuous variables were compared using Kruskal–Wallis tests, whereas categorical variables were compared using χ^2^ tests or Fisher's exact tests where appropriate. These analyses were performed to determine whether differences in baseline maternal or neonatal characteristics were associated with feeding practices. Because feeding practices may have differed according to maternal health and neonatal vulnerability, additional multivariable regression models were fitted including maternal health status as an additional covariate. Separate models further examined maternal influenza during pregnancy and maternal influenza occurring during pandemic‐period pregnancies. These analyses were intended to assess the robustness of the observed feeding‐related associations after accounting for indicators of maternal vulnerability and illness.

Sensitivity analyses were performed by repeating all primary regression models with adjustment for birthweight instead of gestational age. This approach allowed assessment of the robustness of observed associations to alternative adjustment strategies for fetal growth and maturity.

To assess potential bias related to the completeness and timing of postnatal weight measurements, additional sensitivity analyses were performed. Maternal and neonatal characteristics were first compared between infants with one versus two available postnatal weight measurements. Infants with both postnatal weights recorded on days 5 and 10 were compared with infants with all other measurement patterns. Continuous characteristics were compared using *t*‐tests and categorical characteristics using χ^2^ tests or Fisher's exact tests, as appropriate. The primary gestational‐age‐adjusted linear and logistic regression models were repeated in the subgroup of infants with both postnatal weights recorded exactly on days 5 and 10, using the same model specifications as in the primary analyses.

Assessment of effect modification: To investigate whether the association between breastfeeding and high neonatal weight loss differed across predefined subgroups, stratified analyses were performed according to prematurity status, low birthweight, neonatal sex, pregnancy exposure to the First World War, pregnancy exposure to the influenza pandemic, and maternal influenza during pregnancy. Separate logistic regression models were fitted within each stratum, and stratum‐specific odds ratios for breastfeeding were extracted. Heterogeneity of breastfeeding associations across strata was formally assessed using Cochran–Q tests based on the stratum‐specific odds ratios and their corresponding confidence intervals.

For all analyses, statistical significance was defined as a two‐sided *p*‐value < 0.05. Results are presented as regression coefficients (β) with 95% confidence intervals for linear models and odds ratios (ORs) with 95% confidence intervals for logistic models.

All statistical analyses were performed using R statistical software (Version 4.3.2). Analytical code and study documentation are publicly available via GitHub.

## 3 Results

### 3.1 Study Population Characteristics

A total of 13 033 historical birth records from the Lausanne Maternity Hospital were initially available for analysis. After excluding stillbirths (*n* = 536, 4.1%), records with missing live‐birth status (*n* = 1, < 0.1%), records with missing birthweight information (*n* = 8, 0.1%), and newborns without postnatal weight measurements (*n* = 352, 2.7%), 12 136 singleton live births remained eligible for analysis. Because the objective of this study was to characterize early neonatal weight loss, observations with weight trajectories that could not be reliably interpreted in relation to this objective were excluded according to predefined data‐cleaning criteria. These observations either showed weight loss exceeding 20% of birthweight (*n* = 88, 0.73%) or weight gain during the available postnatal observation period (*n* = 309, 2.55%). Overall, 397 observations (3.27% of eligible singleton live births) met these exclusion criteria and were removed from the analytical dataset. The final analytical sample therefore comprised 11 739 singleton live births with available neonatal follow‐up weight measurements, representing 90.1% of the initial study population (Figure 1). Characteristics of eligible live‐born infants excluded because of non‐evaluable weight trajectories are shown in Table S1. Compared with infants included in the analytical cohort, excluded infants had lower mean birthweight (3031 g vs. 3225 g, *p* < 0.001) and lower mean gestational age (39.1 vs. 39.4 weeks, *p* < 0.001), and they were more frequently preterm (10.1% vs. 6.5%, *p* = 0.007) or low birthweight (15.4% vs. 6.3%, *p* < 0.001). Feeding mode also differed between included and excluded infants (*p* = 0.009). Maternal age, neonatal sex, and World War I exposure did not differ substantially between groups.

**FIGURE 1:**
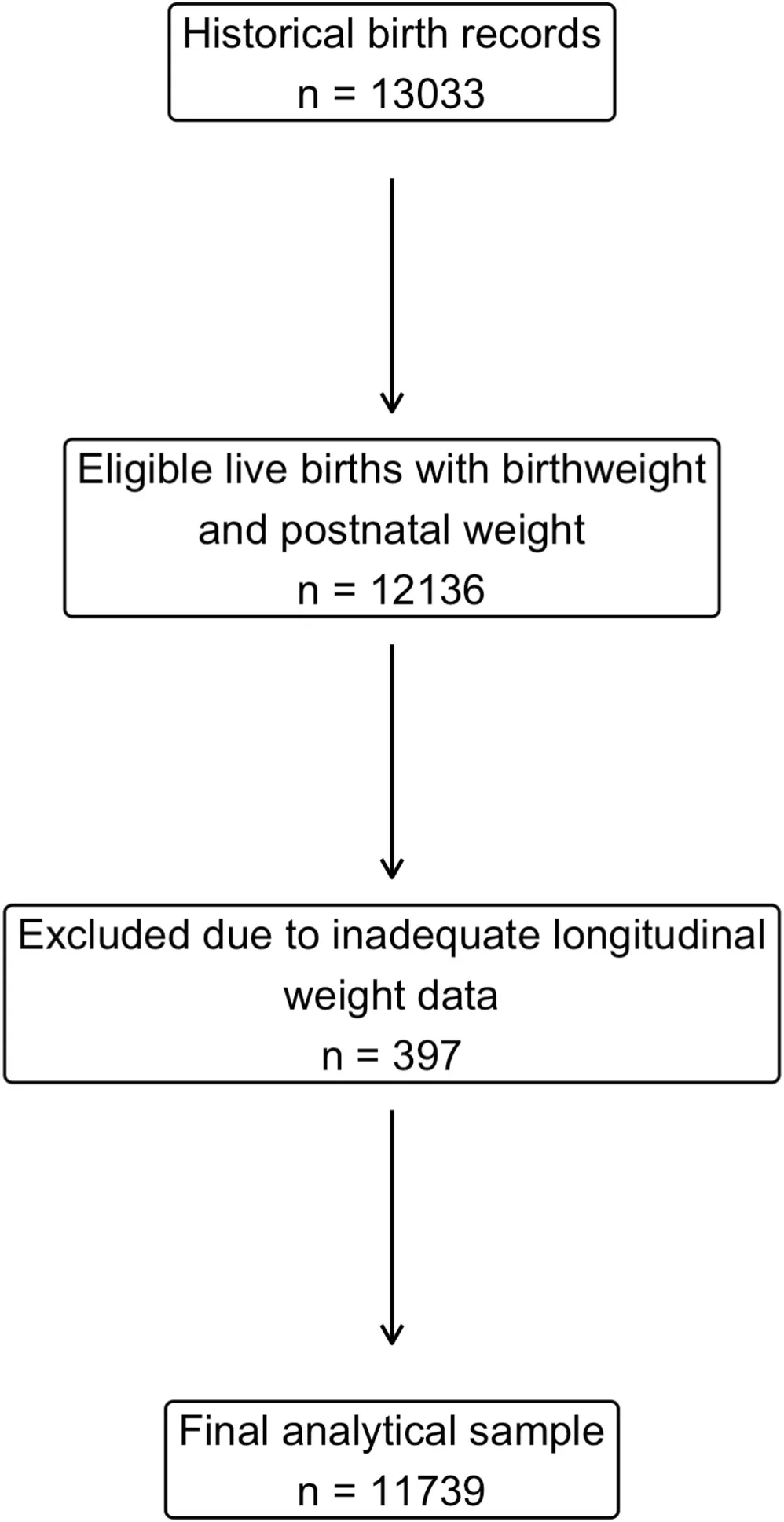
Lausanne maternity hospital study population flowchart (1905–1924).

Maternal, neonatal, and obstetric characteristics of the study population are summarized in Table 1. Mean maternal age was 28.1 years (SD 6.3), and 37.2% were first parities. Mean gestational age was 39.4 weeks (SD 1.6), indicating that the majority of newborns (93.5%) were delivered at term. Mean birthweight was 3225 g (SD = 493 g). Sex distribution was balanced, with 50.7% female and 49.2% male newborns. Breastfeeding represented the predominant feeding practice during the postpartum period in the hospital, accounting for 77.9% of newborns, whereas 13.8% received mixed feeding and 6.4% artificial feeding.

**TABLE 1:** Maternal, neonatal, and obstetric characteristics of the analytical study population and distribution of historical exposure variables (*n* = 11 739).

Characteristic	Value
Continuous characteristics	
Maternal age, years	28.1 (6.3)
Gestational age, weeks	39.4 (1.6)
Birthweight, g	3225 (493)
Categorical characteristics	
Parity	
Parity = 1	4364 (37.2%)
Parity = 2	2618 (22.3%)
Parity = 3	1531 (13.0%)
Parity ≥ 4	3226 (27.5%)
Historical exposures	
Pregnancy during World War I	2975 (25.3%)
Pregnancy during influenza pandemic	2012 (17.1%)
Maternal influenza during pregnancy	288 (2.5%)
Neonatal characteristics	
Male sex	5772 (49.2%)
Female sex	5946 (50.7%)
Preterm birth (< 37 weeks)	768 (6.5%)
Low birth weight (< 2500 g)	736 (6.3%)
Neonatal mortality within 28 days, *n* (%)	114 (0.97%)
Feeding characteristics	
Artificial	754 (6.4%)
Breastfeeding	9142 (77.9%)
Mixed	1625 (13.8%)
Others	183 (1.6%)
Outcome	
Maximum neonatal weight loss, % (mean, SD)	6.73 (3.51)
Maximum neonatal weight loss, % (median, IQR)	6.32 (4.53)
Excessive neonatal weight loss (> 10%)	1971 (16.8%)

*Note:* Continuous variables are presented as mean (SD) or median (IQR). Categorical variables are presented as *n* (%).

### 3.2 Completeness and Timing of Postnatal Weight Assessments

Postnatal weight measurements were mostly complete. Of the 11 739 infants in the analytical cohort, 11 618 (98.97%) had two available postnatal weight measurements and 121 (1.03%) had only one. Among infants with non‐missing timing information for the respective measurement, the first postnatal weight was recorded on day 5 in 11 194 infants (95.42%), while the second was recorded on day 10 in 9959 infants (85.71%) (Figure S7).

Infants with only one available postnatal weight represented a clinically different subgroup. Compared with infants with two measurements, they had lower mean gestational age (38.01 vs. 39.42 weeks) and birthweight (2880 vs. 3229 g), and higher frequencies of preterm birth (24.0% vs. 6.4%), low birthweight (24.0% vs. 6.1%), and neonatal mortality within 28 days (44.6% vs. 0.52%; all *p* < 0.001) (Table S8).

A total of 9779 infants (83.3% of the analytical cohort) had both postnatal weights recorded exactly on days 5 and 10. Compared with infants with other measurement patterns, these infants had slightly higher mean gestational age (39.45 vs. 39.20 weeks) and birthweight (3238 vs. 3159 g), and lower frequencies of preterm birth (6.1% vs. 8.5%), low birthweight (5.9% vs. 8.2%), and neonatal mortality within 28 days (0.43% vs. 3.67%) (Table S9). Maximum observed neonatal weight loss occurred at the first postnatal assessment in 86.6% of infants and at the second assessment in 13.4%.

### 3.3 Distribution of Neonatal Weight Loss

Maximum observed neonatal weight loss (as expressed as a percentage of birthweight) during the first days of life showed interindividual variability. Mean neonatal weight loss was 6.7% (SD 3.5). The distribution of maximum observed neonatal weight loss is shown in Figure 2. Overall, 1971 newborns (16.8%) experienced high neonatal weight loss exceeding 10% of birthweight. Weight loss differed substantially according to feeding type (Figure 3): Breastfed newborns demonstrated the lowest mean weight loss (average 6.3%, SD 3.4), whereas artificially fed newborns showed a mean weight loss of 7.5% (SD 3.6). Similarly, high weight loss occurred in 13.5% of breastfed newborns, compared with 23.1% of artificially fed newborns and 30.2% of mixed‐fed newborns. In descriptive analyses by historical period, average weight loss was 6.5% during the pre‐war period, 7.0% during the First World War, 7.1% during the influenza/post‐war period, and 6.6% during the post‐crisis period. The highest mean neonatal weight loss was observed during the influenza/post‐war period, whereas values during the post‐crisis years returned to levels comparable to those observed before the First World War (Figures S2 and S5). Likewise, the proportion of neonates experiencing high weight loss (> 10%) appeared higher during the First World War and influenza/post‐war periods than during the pre‐war and post‐crisis periods.

**FIGURE 2:**
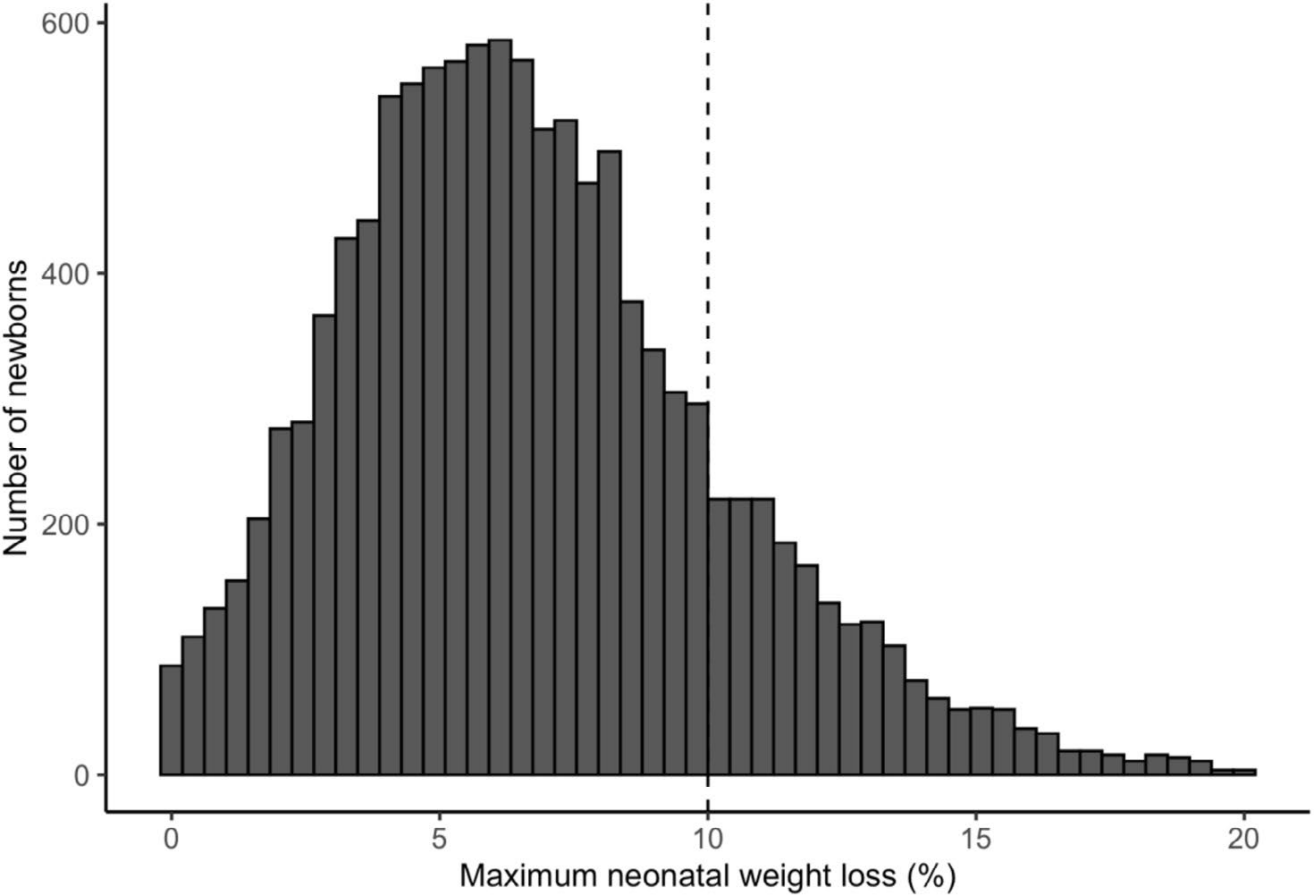
Distribution of maximum observed neonatal weight loss as a percentage of birth weight during the first days of life. The vertical dashed reference line indicates the threshold of high neonatal weight loss, which is defined as a loss of > 10% of birth weight.

**FIGURE 3:**
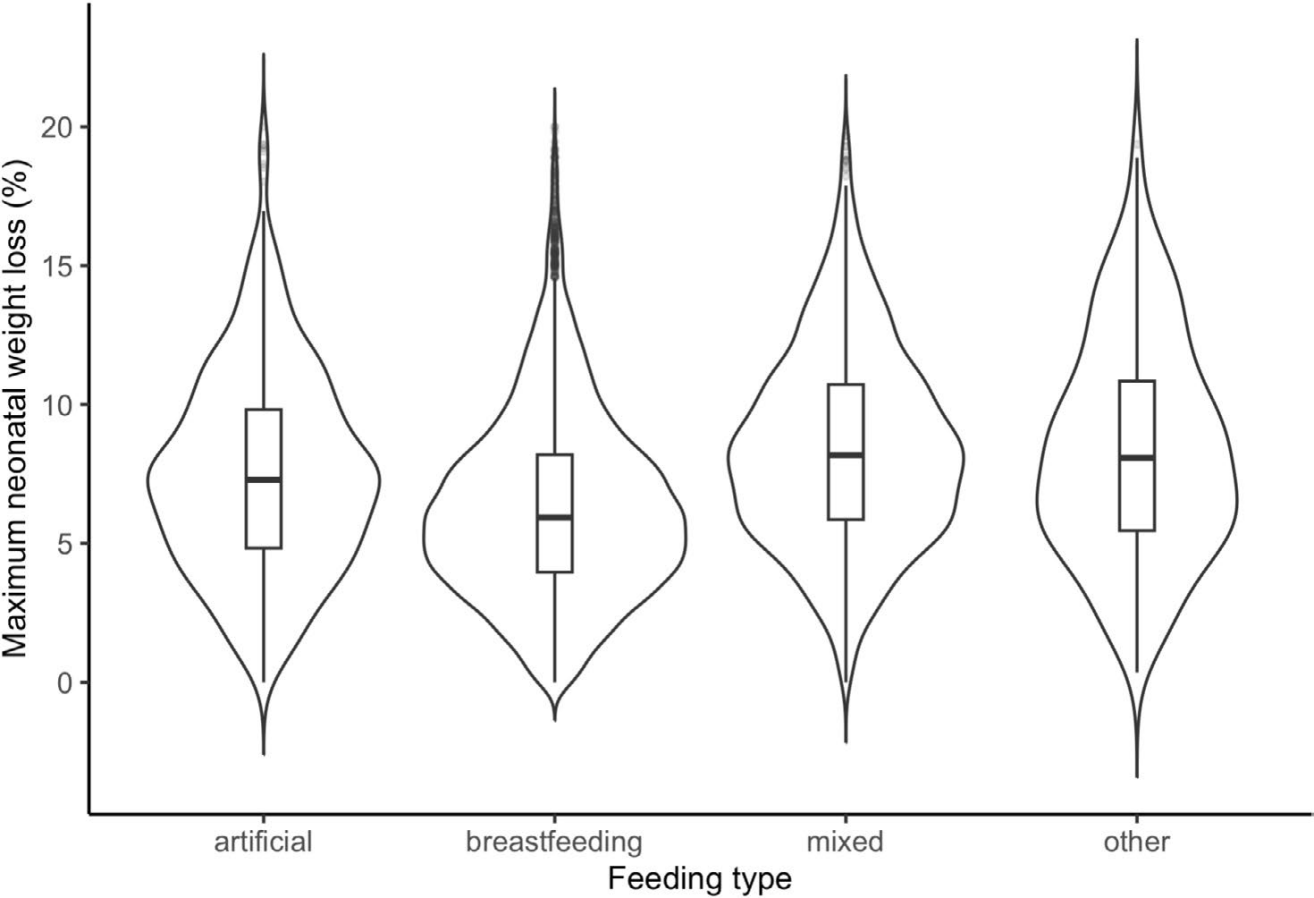
Violin plots of the maximum percent of birth weight lost by neonates according to feeding type during the postpartum period.

### 3.4 Main Multivariable Analyses of Neonatal Weight Loss (Gestational‐Age‐Adjusted)

In the main gestational‐age‐adjusted linear regression model, feeding mode was strongly associated with maximum observed neonatal weight loss (Table 2). Compared with artificially fed newborns, breastfed newborns had substantially lower maximum weight loss (β = −1.07 percentage points, 95% CI −1.32 to −0.81), whereas mixed feeding was associated with higher maximum weight loss (β = 0.94 percentage points, 95% CI 0.65 to 1.23), after adjustment for gestational age, maternal age, parity, neonatal sex, and historical exposure variables. Compared with pregnancies not overlapping the respective exposure period, pregnancy overlapping the influenza pandemic was associated with greater neonatal weight loss (β = 0.44 percentage points, 95% CI 0.27 to 0.60), whereas pregnancy overlapping the First World War showed a small association (β = 0.11 percentage points, 95% CI −0.03 to 0.25), with the confidence interval including the null.

**TABLE 2:** Multivariable linear regression model evaluating associations with maximum observed neonatal weight loss (%) in the gestational‐age‐adjusted main model.

Term	Estimate	Standard error	Statistic	*p*	conf.low	conf.high
Intercept	18.542	0.785	23.625	< 0.001	17.004	20.08
Pregnancy overlapping World War I: yes versus no	0.11	0.072	1.525	0.127	−0.032	0.253
Pregnancy overlapping influenza pandemic: yes versus no	0.438	0.084	5.233	< 0.001	0.274	0.602
Feeding mode: breastfeeding versus artificial feeding	−1.067	0.129	−8.283	< 0.001	−1.319	−0.814
Feeding mode: mixed feeding versus artificial feeding	0.938	0.149	6.275	< 0.001	0.645	1.231
Feeding mode: other versus artificial feeding	0.75	0.28	2.68	0.007	0.201	1.299
Gestational age, weeks	−0.295	0.019	−15.174	< 0.001	−0.333	−0.257
Male sex versus female	−0.088	0.063	−1.413	0.158	−0.211	0.034
Parity	−0.069	0.015	−4.592	< 0.001	−0.098	−0.039
Maternal age, years	0.023	0.006	3.682	< 0.001	0.011	0.036

*Note:* Estimates are shown for infant feeding mode, historical pregnancy exposures, gestational age, neonatal sex, parity, and maternal age. Reference categories were artificial feeding for feeding mode, no exposure for World War I and influenza pandemic exposure, and female sex for neonatal sex.

Male and female newborns had similar maximum observed neonatal weight loss in the main model. In the corresponding gestational‐age‐adjusted logistic regression model for high neonatal weight loss > 10% (Table 3), breastfeeding was associated with substantially lower odds of high weight loss compared with artificial feeding (OR = 0.56, 95% CI 0.47 to 0.68). In contrast, mixed feeding was associated with higher odds of high weight loss (OR = 1.52, 95% CI 1.24 to 1.87), as was the “other” feeding category (OR = 1.45, 95% CI 1.00 to 2.09).

**TABLE 3:** Multivariable logistic regression model evaluating associations with high neonatal weight loss (> 10%) in the gestational‐age‐adjusted main model.

Term	Odds ratio	Standard error	Statistic	*p*	ci_low_	ci_high_
Intercept	687.269	0.562	11.619	< 0.001	228.421	2070.951
Pregnancy overlapping World War I: yes versus no	1.087	0.058	1.452	0.146	0.971	1.216
Pregnancy overlapping influenza pandemic: yes versus no	1.117	0.066	1.679	0.093	0.981	1.271
Feeding mode: breastfeeding versus artificial feeding	0.56	0.094	−6.153	< 0.001	0.467	0.675
Feeding mode: mixed feeding versus artificial feeding	1.519	0.105	4	< 0.001	1.24	1.868
Feeding mode: other versus artificial feeding	1.454	0.188	1.993	0.046	1.001	2.092
Gestational age, weeks	0.819	0.014	−14.281	< 0.001	0.797	0.842
Male sex versus female	0.92	0.051	−1.644	0.1	0.832	1.016
Parity	0.976	0.012	−1.994	0.046	0.953	0.999
Maternal age, years	1.004	0.005	0.703	0.482	0.994	1.014

*Note:* Odds ratios are shown for infant feeding mode, historical pregnancy exposures, gestational age, neonatal sex, parity, and maternal age. Reference categories were artificial feeding for feeding mode, no exposure for World War I and influenza pandemic exposure, and female sex for neonatal sex.

Overall, feeding mode showed by far the strongest association with neonatal weight loss. Pregnancy overlapping the influenza pandemic was associated with higher maximum observed neonatal weight loss compared with pregnancies not overlapping the influenza pandemic, whereas evidence for an association with high neonatal weight loss was weaker. There was no clear evidence that the odds of high neonatal weight loss differed by neonatal sex in the main gestational‐age‐adjusted model (OR = 0.92, 95% CI 0.83 to 1.02; Figure 4).

**FIGURE 4:**
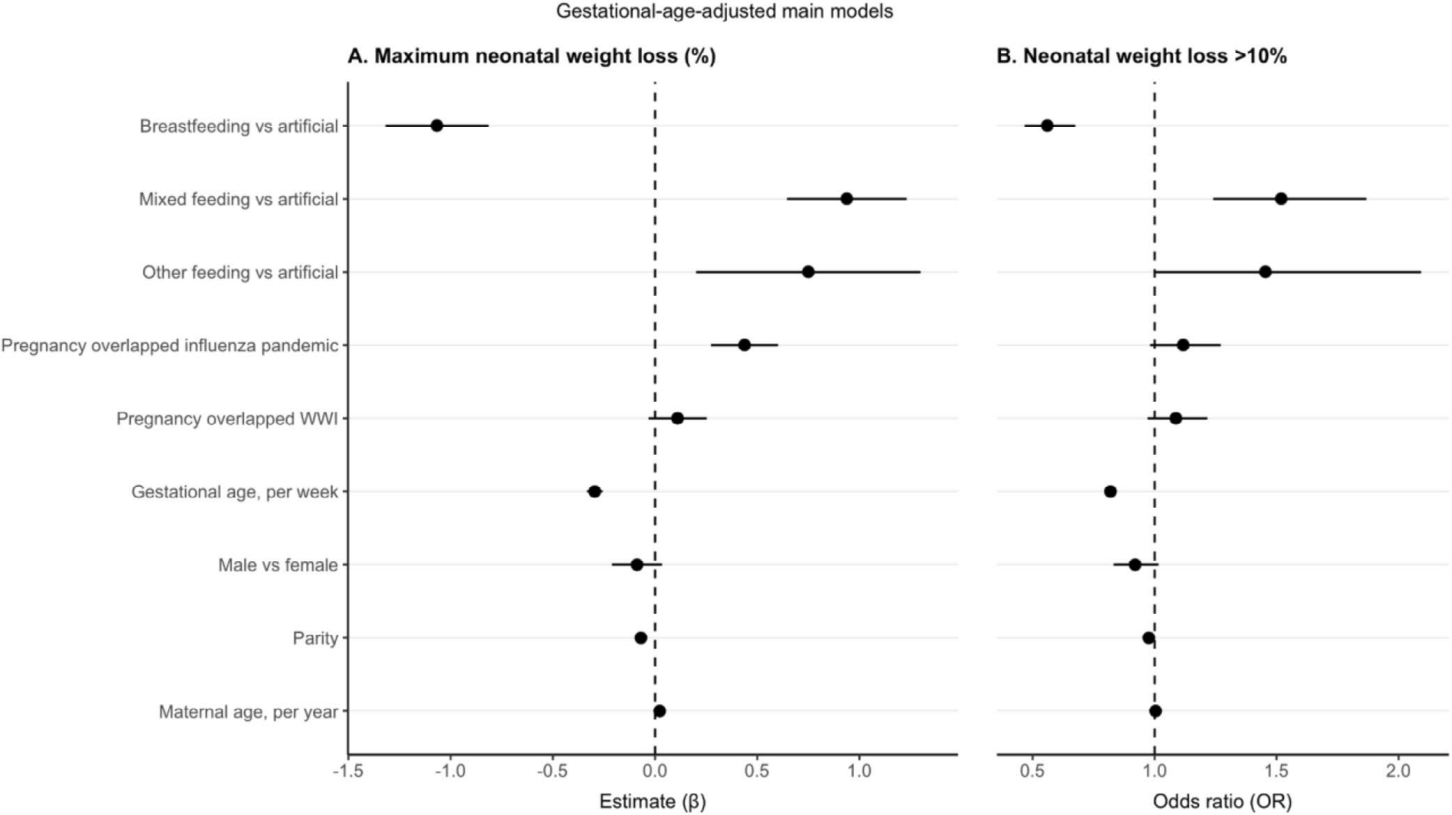
Adjusted associations of infant feeding mode, maternal characteristics, neonatal factors, and historical crisis exposures with neonatal weight loss. Panel A shows regression coefficients (β) with 95% confidence intervals for maximum observed neonatal weight loss (%); Panel B shows odds ratios (OR) with 95% confidence intervals for high neonatal weight loss (> 10%).

Maternal and neonatal characteristics differed significantly across feeding categories (Table S2). Breastfeeding was generally more common among newborns with more favorable maternal and neonatal characteristics, whereas artificial and mixed feeding were more frequently observed among clinically vulnerable mother–infant pairs. Artificially fed newborns had lower mean gestational age (39.1 vs. 39.5 weeks, *p* < 0.001) and lower mean birthweight (3127 vs. 3238 g, *p* < 0.001) than breastfed newborns. Consistent with these differences, low birthweight and preterm birth were more common among artificially fed infants (11.3% and 11.1%, respectively) than among breastfed infants (5.3% and 5.7%, respectively; all *p* < 0.001). Mothers of artificially fed and mixed‐fed newborns were older and had higher parity than mothers who breastfed. Poor maternal health status was more common among artificially fed and mixed‐fed infants than among breastfed infants (all *p* < 0.001). Maternal influenza during pregnancy and maternal influenza occurring during the pandemic period were also unevenly distributed across feeding categories. Both exposures were more frequent among mixed‐fed and other‐fed newborns than among breastfed or artificially fed newborns (all *p* < 0.001). In contrast, neonatal sex distribution did not differ substantially according to feeding mode (*p* = 0.309).

### 3.5 Maternal Influenza and Pandemic‐Related Exposure Analyses

Additional analyses examined whether maternal influenza exposure during pregnancy was associated with neonatal weight loss independently of feeding mode and other covariates. Maternal influenza during pregnancy was associated with an average increase of 0.42 percentage points in maximum observed neonatal weight loss (95% CI 0.03 to 0.82) compared with pregnancies in which the mother did not have documented influenza during pregnancy (Table S3). Despite inclusion of maternal influenza in the model, the associations between feeding mode and neonatal weight loss remained largely unchanged. Compared with artificial feeding, breastfeeding was associated with 1.06 percentage points lower neonatal weight loss (95% CI −1.32 to −0.81), whereas mixed feeding was associated with 0.96 percentage points higher neonatal weight loss (95% CI 0.67 to 1.26). A second model evaluated a combined exposure variable representing maternal influenza during pregnancy occurring within the influenza pandemic period (Table S4). This exposure showed a similar association with an estimated increase of 0.41 percentage points in neonatal weight loss (95% CI 0.00 to 0.82). Overall, these findings suggest that maternal influenza exposure was not a strong confounder of the association between feeding mode and neonatal weight loss, but that it was associated with greater neonatal weight loss.

### 3.6 Sensitivity Tests, Stratified Analyses, and Effect Modification

Sensitivity analyses were performed using birthweight adjustment instead of gestational age adjustment. Overall, results were highly consistent with those observed in the main gestational‐age‐adjusted analyses (Tables S5 and S6). These findings indicate that the observed associations between feeding mode, pandemic exposure, and neonatal weight loss were robust to alternative adjustment strategies.

Furthermore, stratified analyses were performed to evaluate whether the association between breastfeeding and high neonatal weight loss differed according to neonatal vulnerability (only term newborns and normal birthweight), sex, or historical crisis exposure status (Table S7).

Across most strata examined, breastfeeding was associated with lower odds of high neonatal weight loss compared with artificial feeding, although estimates were less precise among preterm and low‐birthweight newborns. Among term newborns, breastfeeding was associated with lower odds of high neonatal weight loss (OR = 0.53, 95% CI 0.44–0.65) compared with artificial feeding, while the corresponding estimate among preterm newborns was less precise (OR = 0.75, 95% CI 0.45–1.26). No evidence of heterogeneity between term and preterm strata was observed according to the Cochran–Q test (*p* = 0.239). Comparable findings were observed when analyses were stratified by low birthweight status. Breastfeeding was associated with lower odds of high neonatal weight loss among normal‐birthweight newborns (OR = 0.53, 95% CI 0.43–0.64), whereas the estimate among low‐birthweight newborns was imprecise (OR = 0.91, 95% CI 0.53–1.57). The protective association of breastfeeding was also observed irrespective of historical exposure status (Figure S1). Breastfeeding was associated with lower odds of high neonatal weight loss among pregnancies occurring both outside and during the influenza pandemic, as well as among pregnancies occurring both outside and during the First World War. Similar findings were observed when analyses were stratified according to maternal influenza during pandemic‐period pregnancy and by neonatal sex. Overall, the association between breastfeeding and lower odds of high neonatal weight loss was consistent across the examined neonatal subgroups and historical exposure periods.

As an additional sensitivity analysis addressing variation in postnatal weight measurement timing, the primary gestational‐age‐adjusted models were repeated among the 9779 infants with both postnatal weight measurements recorded exactly on days 5 and 10 (Table S10). In the linear model, breastfeeding remained associated with lower maximum neonatal weight loss compared with artificial feeding (β = −1.147, 95% CI −1.424 to −0.871; *p* < 0.001), whereas mixed feeding remained associated with greater weight loss (β = 0.860, 95% CI 0.543 to 1.176; *p* < 0.001). The association with pregnancy overlapping the influenza pandemic was also similar to the primary analysis (β = 0.388, 95% CI 0.218 to 0.558; *p* < 0.001). There was no clear evidence of an association between World War I exposure and maximum neonatal weight loss (β = 0.107, 95% CI −0.042 to 0.256; *p* = 0.158). In the corresponding logistic model, breastfeeding remained associated with lower odds of neonatal weight loss > 10% (OR = 0.530, 95% CI 0.434–0.650; *p* < 0.001), and mixed feeding with higher odds (OR = 1.453, 95% CI 1.167–1.817; *p* < 0.001). Overall, restriction to standardized day‐5 and day‐10 measurements did not materially alter the principal feeding associations observed in the primary analyses.

## 4 Discussion

In this historical cohort of 11 739 neonates born between 1905 and 1924, feeding mode emerged as the strongest determinant of neonatal weight loss. Breastfeeding was consistently associated with lower maximum observed neonatal weight loss and lower odds of high weight loss, whereas mixed feeding was associated with higher weight loss across all analyses. These associations remained highly consistent across the main gestational‐age‐adjusted models and birthweight‐adjusted sensitivity models, indicating that feeding‐related effects were robust to alternative modeling strategies. In addition to feeding mode, maternal influenza exposure and pregnancies overlapping the 1918–1920 influenza pandemic were associated with increased neonatal weight loss. There was no clear evidence of an independent association between First World War exposure and neonatal weight loss after adjustment for feeding mode and perinatal characteristics. However, the magnitude of the feeding‐related associations substantially exceeded that of the historical crisis‐related exposures, highlighting feeding mode as the dominant correlate of neonatal weight loss in this cohort.

### 4.1 Feeding Mode and Neonatal Weight Loss

Breastfeeding was consistently associated with lower neonatal weight loss and lower odds of high neonatal weight loss, whereas mixed feeding was associated with greater neonatal weight loss. The strong association between feeding mode and neonatal weight loss is broadly consistent with contemporary neonatal literature, which identifies infant feeding as one of the most important determinants of early postnatal weight trajectories. Several modern studies have reported significant associations between feeding practices and both physiological and high neonatal weight loss (Dewey 2001; DiTomasso and Cloud 2019; Kellams, Harrel, Omage, Gregory, and Rosen‐Carole 2017; Macdonald et al. 2003; Miyoshi et al. 2020; Regnault et al. 2011). However, the direction of the association observed in the present study differs from that reported in many contemporary cohorts. Modern studies frequently describe greater early physiological weight loss among exclusively breastfed newborns, particularly during the establishment of lactation and the transition to effective milk transfer (Dewey 2001; DiTomasso and Cloud 2019; Macdonald et al. 2003; Miyoshi et al. 2020; Regnault et al. 2011). In contrast, breastfed infants in our historical cohort experienced lower neonatal weight loss than artificially fed infants. From a human‐biological perspective, lactation represents an important component of maternal investment, marking the transition from prenatal placental provisioning to postnatal transfer of maternal resources to the offspring (Dib et al. 2023; Miller et al. 2025; Wells 2010).

Recent work has emphasized that human milk should be considered a dynamic and variable component of the human phenotype rather than a uniform nutritional exposure, with its characteristics embedded within maternal, infant, and ecological contexts (Miller et al. 2025; Quinn 2021). Lactation can likewise be viewed as a flexible form of maternal investment, for which mothers may draw on dietary intake, energy conservation, and body reserves to meet the energetic demands of milk production (Piperata 2009). Consistent with the importance of the broader maternal–infant context, a recent study of healthy term breastfed infants showed that postnatal weight gain during the first month was associated with maternal BMI, mode of delivery, birthweight, and neonatal jaundice (Aydın and Yalçin 2024).

One possible explanation is that artificial feeding practices in the early twentieth century differed substantially from contemporary formula feeding (Mepham 1993; Vögele et al. 2013). Artificial feeding may not have provided the same nutritional support as modern infant formula and may therefore have been associated with less favorable early postnatal weight trajectories. Artificial feeding practices in the early twentieth century differed substantially from those of today (Mepham 1993; Vögele et al. 2013). Commercial infant formulas were not yet widely available, and artificial feeding generally consisted of animal milk or other milk substitutes prepared according to contemporary infant‐feeding practices. Consequently, feeding categories recorded in historical maternity records should not be assumed to be directly comparable with contemporary infant feeding practices.

Taken together, these findings suggest that feeding mode played a central role in shaping neonatal weight trajectories in this historical population. They are compatible with a broader human‐biological interpretation in which the establishment of postnatal feeding forms part of the transition from prenatal maternal provisioning to postnatal nutritional dependence. The observed association between breastfeeding and lower neonatal weight loss highlights the importance of interpreting infant feeding within its specific historical context and suggests that the relationship between feeding mode and neonatal weight loss may have differed from that observed in modern clinical settings.

### 4.2 Feeding Practices and Confounding by Indication

An important consideration when interpreting the observed feeding‐related associations is that feeding mode was unlikely to be randomly distributed within the cohort. Artificially fed infants had lower birthweight, lower gestational age, and higher frequencies of low birthweight and prematurity than breastfed infants. In addition, poorer maternal health was more common among mothers whose infants received artificial or mixed feeding. These findings suggest that feeding mode may have reflected underlying maternal and neonatal vulnerability in addition to nutritional exposure itself.

These patterns can also be considered within the maternal capital framework, in which aspects of maternal phenotype and condition represent resources available for investment in offspring (Dib et al. 2023; Wells 2010, 2025). Maternal condition may therefore be related both to postnatal maternal investment and to offspring condition, potentially contributing to the observed relationships between maternal vulnerability, feeding practices, and neonatal outcomes. Importantly, the variables available in our historical records do not constitute a direct measure of maternal capital; this framework is therefore used as an interpretive perspective rather than as a mechanism directly tested in the present study.

This interpretation is consistent with historical infant feeding practices. Breastfeeding remained the dominant mode of infant feeding throughout much of Europe during the late nineteenth and early twentieth centuries, whereas artificial feeding was often introduced when breastfeeding was not possible because of maternal illness, lactation difficulties, neonatal weakness, or other medical complications. Artificial feeding therefore frequently represented a response to adverse maternal or neonatal circumstances rather than a purely elective feeding choice (Mepham 1993; Vögele et al. 2013). Consequently, feeding mode may have acted as both a nutritional exposure and a marker of vulnerability. Infants who were already at increased biological risk may have been more likely to receive artificial or mixed feeding and may also have been more susceptible to greater postnatal weight loss. Confounding by indication therefore remains a plausible explanation for part of the observed association between feeding mode and neonatal weight loss. However, the associations between feeding mode and neonatal weight loss remained evident after adjustment for maternal health status and across alternative models adjusting for either gestational age or birthweight, and other relevant covariates, and in analyses stratified by preterm birth or low birthweight status. Although residual confounding cannot be excluded, these findings suggest that differences in vulnerability alone are unlikely to fully explain the observed associations. Taken together, feeding mode in this historical cohort likely reflected both infant nutrition and underlying maternal‐neonatal vulnerability. The observed associations should therefore be interpreted as the combined result of nutritional and clinical factors rather than as purely causal effects of feeding type.

### 4.3 Prenatal Adversity, Maternal Influenza and Neonatal Adaptation

In addition to feeding‐related factors, maternal influenza exposure during pregnancy and pregnancies overlapping the 1918–1920 influenza pandemic were associated with increased neonatal weight loss. Although these associations were smaller than those observed for feeding mode, they suggest that prenatal exposures may have contributed to variation in early neonatal weight trajectories. These findings are compatible with developmental plasticity frameworks, in which adverse prenatal environments may influence fetal growth and physiological development, with potential consequences extending into the neonatal period (Wells 2025). Previous studies have demonstrated associations between maternal infection, nutritional deprivation, and altered fetal growth, birth outcomes, and later‐life health (Barker 1990; Roseboom et al. 2001; Scholte et al. 2015). From a human‐biological perspective, developmental plasticity provides a framework for considering how maternally mediated nutritional and physiological signals during fetal life may shape developmental trajectories and subsequent responses to the postnatal environment (Kuzawa 2005).

Historical studies of prenatal adversity, including the Dutch Hunger Winter and the 1918 influenza pandemic, have further shown that gestational exposures may have lasting effects on offspring health and development (Beach et al. 2022; Roseboom et al. 2001; Scholte et al. 2015). Several biological mechanisms may explain the observed associations. Maternal influenza infection may influence fetal development through inflammatory pathways, placental dysfunction, altered nutrient transfer, or impaired fetal growth (Weckman et al. 2019). Such processes could affect neonatal physiological adaptation after birth and potentially contribute to greater postnatal weight loss. More broadly, pregnancies occurring during the influenza pandemic may have been exposed not only to infectious disease but also to nutritional, social, and psychological stressors that could have influenced fetal development and neonatal adaptation.

Our findings are also consistent with recent historical studies from Switzerland. Le Vu et al. reported associations between maternal influenza‐like illness and adverse neonatal outcomes during the 1918 pandemic using the same dataset, while additional work has suggested that prenatal infectious exposures may influence fetal growth patterns and neonatal development (Le Vu et al. 2024, 2025). The present findings extend this literature by suggesting that prenatal infectious exposures may also be associated with early postnatal weight trajectories. Nevertheless, the observational nature of the study precludes causal inference. Because maternal influenza was identified from physician‐recorded diagnoses, the observed association should be interpreted cautiously, as incomplete confirmation of influenza cases cannot be excluded. The observed associations should therefore be interpreted as compatible with developmental origins hypotheses rather than as direct evidence of causal prenatal programming. Taken together, these findings suggest that prenatal infectious exposures may have contributed to altered neonatal adaptation, reflected by increased neonatal weight loss during the early postnatal period.

### 4.4 Historical Crisis Periods: Influenza Pandemic Versus World War I

Previous historical studies from Switzerland and other European settings have linked wartime conditions, food shortages, and infectious disease outbreaks to changes in birthweight, placental characteristics, and other indicators of neonatal health (Butie et al. 2020; Floris and Staub 2025; Floris et al. 2016; Salvisberg et al. 2023; Sonderegger and Tscherrig 2016). Similarly, studies using historical maternity records have reported adverse effects of the First World War and related societal disruptions on neonatal anthropometric outcomes (Butie et al. 2020; Floris et al. 2016; Salvisberg et al. 2023). The present findings only partially align with this literature. While pregnancies occurring during the influenza pandemic showed increased neonatal weight loss, there was no clear independent association with pregnancy overlap with the First World War. One possible explanation is that pandemic‐related exposures exerted more direct biological effects through maternal infection and inflammation, whereas wartime influences were likely mediated through broader social and nutritional pathways. This contrast may also be viewed in the context of maternal capital. Maternal physiological and nutritional resources may buffer the fetus against some external stressors, whereas maternal infection may affect fetal development more directly through changes in maternal physiology. This may partly explain why World War I exposure showed only a small association with neonatal weight loss, while the association was stronger during the influenza pandemic.

The historical context of Switzerland may also be relevant when interpreting these findings. Although the country experienced substantial economic disruption, food insecurity, and social strain during the First World War, it remained militarily neutral and was not exposed to the widespread destruction experienced in many neighboring countries (Floris and Staub 2025; Krämer et al. 2016; Sonderegger and Tscherrig 2016). Consequently, wartime conditions may have been insufficient to produce an independent effect on neonatal weight loss once maternal, neonatal, and feeding‐related factors were taken into account. An additional consideration is that pandemic‐period exposure may represent a composite marker encompassing infectious, nutritional, social, and psychological stressors occurring simultaneously during the late war and immediate post‐war years. From a human‐biological perspective, the pandemic period may therefore be better interpreted as a broader ecological stress context rather than as a proxy for influenza infection alone. The observed associations should therefore not be interpreted as reflecting the biological effects of influenza infection alone. Taken together, these findings suggest that pandemic‐related exposures were more strongly associated with neonatal weight loss than World War I exposure itself, highlighting the potential importance of infectious and societal stressors for neonatal adaptation in historical populations.

### 4.5 Neonatal Weight Loss as a Marker of Neonatal Adaptation in Historical Populations

Neonatal weight loss may be conceptualized as a short‐term phenotype occurring at the transition between prenatal development and postnatal nutritional provisioning. It may therefore reflect aspects of neonatal physiological adaptation after birth while also being influenced by the establishment and mode of postnatal feeding. In historical populations, neonatal weight trajectories may provide information complementary to conventional birth anthropometry by capturing aspects of adaptation occurring after delivery. The protective association between breastfeeding and lower neonatal weight loss was observed consistently across all examined neonatal subgroups. Cochran–Q heterogeneity tests provided no evidence that the association between breastfeeding and high neonatal weight loss differed across prematurity status, birthweight category, neonatal sex, historical crisis exposure, or maternal influenza exposure during pregnancy. These findings suggest that the association between feeding mode and neonatal weight loss was broadly similar across clinically and historically vulnerable neonatal populations. Nevertheless, the clinical interpretation of proportional weight loss may differ between neonatal populations. Similar percentage weight losses may impose a greater physiological burden in vulnerable infants, particularly those born preterm or with low birthweight, because of their lower metabolic reserves and reduced physiological capacity to compensate for postnatal nutritional deficits. Consequently, statistical consistency across subgroups should not necessarily be interpreted as implying equal clinical relevance across all neonatal populations. Taken together, these findings suggest that the association between breastfeeding and lower neonatal weight loss was broadly consistent across vulnerable neonatal populations, further supporting the robustness of the observed feeding‐related effects.

### 4.6 Strengths and Limitations

This study has several strengths. First, it is based on a large historical cohort comprising more than 11 000 neonates with serial weight measurements, with approximately 99% having two available measurements, allowing detailed assessment of early neonatal weight trajectories. Second, the availability of detailed feeding information enabled evaluation of feeding‐related determinants of neonatal weight loss in a historical setting. Third, the study was conducted within a unique period encompassing both the First World War and the 1918–1920 influenza pandemic, providing an opportunity to investigate the influence of major societal and infectious stressors on neonatal outcomes. In addition, the availability of maternal, neonatal, and perinatal information allowed adjustment for a broad range of potential confounders. Finally, the use of multiple sensitivity analyses strengthened the robustness of the findings.

Several limitations should also be considered. The observational design precludes causal inference and leaves the possibility of residual confounding. In particular, confounding by indication may have influenced the observed associations between feeding mode and neonatal weight loss, as feeding allocation was unlikely to be random. Furthermore, the use of historical hospital records inevitably entails limitations related to data completeness, measurement accuracy, and potential misclassification of exposures and outcomes. Postnatal weight measurements were primarily available around days 5 and 10 rather than continuously across the first postnatal days. Consequently, the true postnatal weight nadir may not have been captured in all infants, and the maximum observed weight loss should be interpreted as an approximation of maximal early neonatal weight loss. In some infants, particularly those with later measurements, weight recovery may already have begun by the time of assessment.

Variation in the timing and completeness of postnatal weight measurements may have introduced selection bias. However, the principal associations were similar when analyses were restricted to infants with standardized day‐5 and day‐10 measurements, suggesting that variation in measurement timing was unlikely to fully account for the observed findings. Residual measurement error related to the historical clinical recording process nevertheless cannot be excluded. The markedly higher neonatal mortality and greater clinical vulnerability among infants with only one available postnatal weight measurement indicate that incomplete follow‐up was not random. However, this group represented only 1.03% of the analytical cohort.

Observations with non‐evaluable weight trajectories were excluded based on predefined biological plausibility criteria. Although these exclusions represented only a small proportion of eligible live‐born infants, excluded infants were more likely to have indicators of neonatal vulnerability, including lower birthweight, lower gestational age, preterm birth, and low birthweight. This may have led to a slight underrepresentation of clinically vulnerable newborns in the analytical cohort. Information on feeding frequency, milk volume, and actual nutritional intake was unavailable, limiting detailed assessment of feeding practices and preventing evaluation of dose‐dependent feeding effects. In addition, the historical records did not provide direct measures of maternal nutritional intake, energy balance, milk production or composition, placental function, or other physiological components relevant to maternal capital. Consequently, the maternal‐investment and developmental‐plasticity frameworks used in this study should be considered interpretive perspectives rather than mechanisms directly tested in the present analyses. In addition, maternal influenza exposure was likely underestimated since only clinically documented cases could be identified. Finally, the cohort originated from a single maternity hospital, which may limit the generalizability of the findings to other historical populations, geographical settings, or healthcare systems.

## 5 Conclusion

In this historical cohort of neonates born between 1905 and 1924, feeding mode emerged as the strongest correlate of neonatal weight loss. Breastfeeding was consistently associated with lower neonatal weight loss, whereas mixed feeding was associated with greater weight loss across multiple analytical approaches. In addition, maternal influenza exposure and pregnancies occurring during the 1918–1920 influenza pandemic were associated with modest increases in neonatal weight loss, while the association with First World War exposure was small and did not provide clear evidence of an independent association. These findings suggest that early neonatal weight trajectories may represent a postnatal phenotype at the intersection of infant physiological adaptation, postnatal nutritional provisioning, and prenatal environmental conditions. Beyond its established clinical relevance, neonatal weight loss may therefore provide information complementary to traditional measures such as birthweight and neonatal anthropometry by capturing aspects of adaptation occurring after delivery. Historical maternity records offer a unique opportunity to investigate how maternal, nutritional, infectious, and broader environmental conditions shape neonatal adaptation during periods of major societal disruption.

## Funding

The authors thank the Swiss National Science Foundation (SNSF, project number 197305, Grantee Kaspar Staub) for providing financial support.

## Ethics Statement

The larger research project, of which this study was a part, was approved by the Zurich Ethic Commission (BASEC Number 2021‐00628).

## Conflicts of Interest

The authors declare no conflicts of interest.

## Supporting information


**Figure S1:** Forest plot showing adjusted associations between breastfeeding and high neonatal weight loss across neonatal vulnerability groups, historical exposure strata, and infant sex.
**Figure S2:** Comparison of maximum observed neonatal weight loss according to historical exposure status, including World War I exposure and pregnancy during the 1918–1920 influenza pandemic.
**Figure S3:** Comparison of adjusted effect estimates from the primary gestational‐age‐adjusted models and birth‐weight‐adjusted sensitivity analyses for neonatal weight loss outcomes.
**Figure S4:** Proportion of newborns experiencing high neonatal weight loss (> 10% of birth weight) according to infant feeding mode.
**Figure S5:** Temporal variation in maximum observed neonatal weight loss among newborns delivered between 1905 and 1924.
**Figure S6:** Temporal trends in the distribution of infant feeding practices across birth years between 1905 and 1924.
**Figure S7:** Distribution of the timing of the first and second postnatal weight measurements in the analytical cohort.
**Table S1:** Characteristics of infants included in the analytical cohort and eligible live‐born infants excluded because of non‐evaluable neonatal weight trajectories, including weight gain (< 0%) or weight loss (> 20%).
**Table S2:** Maternal, neonatal, and obstetric characteristics stratified by infant feeding mode, including demographic factors, pregnancy characteristics, and indicators of neonatal vulnerability.
**Table S3:** Gestational‐age‐adjusted multivariable linear regression analysis evaluating the association between documented maternal influenza during pregnancy and maximum observed neonatal weight loss.
**Table S4:** Multivariable linear regression analysis evaluating the association between documented maternal influenza occurring during pandemic‐period pregnancy and maximum observed neonatal weight loss.
**Table S5:** Birthweight‐adjusted sensitivity analysis examining associations between infant feeding mode, maternal and neonatal characteristics, historical exposures, and maximum observed neonatal weight loss (%).
**Table S6:** Birthweight‐adjusted sensitivity analysis examining associations between feeding mode, maternal and neonatal characteristics, historical crisis exposures, and high neonatal weight loss (> 10%).
**Table S7:** Stratified logistic regression analyses of the association between breastfeeding and high neonatal weight loss across neonatal vulnerability groups, infant sex, and historical exposure strata, with Cochran–Q heterogeneity tests.
**Table S8:** Comparison of maternal and neonatal characteristics between infants with one versus two available postnatal weight measurements.
**Table S9:** Comparison of maternal and neonatal characteristics between infants with both postnatal weight measurements recorded exactly on days 5 and 10 and infants with other measurement patterns.
**Table S10:** Sensitivity analyses restricted to infants with both postnatal weight measurements recorded exactly on days 5 and 10. Gestational‐age‐adjusted linear and logistic regression models were repeated using the same model specifications as in the primary analyses.

## Data Availability

The data that support the findings of this study are openly available in Github at https://github.com/antonnag/Neonatal‐Weight‐Loss‐in‐Historical‐Swiss‐Maternity‐Hospital‐Records‐.
