# Supplementary material for: Determinants of Early Neonatal Weight Loss in Historical Swiss Maternity Hospital Records
Source: Am J Hum Biol. 2026 Oct 20;38(10):e70349. doi: 10.1002/ajhb.70349 (PMC13684055; doi:10.1002/ajhb.70349)
Supplement: Supplementary file 1 [file AJHB-38-e70349-s001.docx]

**SUPPLEMENTARY MATERIAL**

**Supplementary Figure S1:** Forest plot showing adjusted associations between breastfeeding and high neonatal weight loss across neonatal vulnerability groups, historical exposure strata, and infant sex.

**Supplementary Figure S2:** Comparison of maximum observed neonatal weight loss according to historical exposure status, including World War I exposure and pregnancy during the 1918–1920 influenza pandemic.

**Supplementary Figure S3:** Comparison of adjusted effect estimates from the primary gestational-age -adjusted models and birth-weight-adjusted sensitivity analyses for neonatal weight loss outcomes.

**Supplementary Figure S4:** Proportion of newborns experiencing high neonatal weight loss (>10% of birth weight) according to infant feeding mode.

**Supplementary Figure S5:** Temporal variation in maximum observed neonatal weight loss among newborns delivered between 1905 and 1924.

**Supplementary Figure S6:** Temporal trends in the distribution of infant feeding practices across birth years between 1905 and 1924.

**Supplementary Figure S7:** Distribution of the timing of the first and second postnatal weight measurements in the analytical cohort.

**Supplementary Table S1:** Characteristics of infants included in the analytical cohort and eligible live-born infants excluded because of non-evaluable neonatal weight trajectories, including weight gain (<0%) or weight loss (>20%).

**Supplementary Table S2:** Maternal, neonatal, and obstetric characteristics stratified by infant feeding mode, including demographic factors, pregnancy characteristics, and indicators of neonatal vulnerability.

**Supplementary Table S3:** Gestational-age-adjusted multivariable linear regression analysis evaluating the association between documented maternal influenza during pregnancy and maximum observed neonatal weight loss.

**Supplementary Table S4:** Multivariable linear regression analysis evaluating the association between documented maternal influenza occurring during pandemic-period pregnancy and maximum observed neonatal weight loss

**Supplementary Table S5:** Birthweight-adjusted sensitivity analysis examining associations between infant feeding mode, maternal and neonatal characteristics, historical exposures, and maximum observed neonatal weight loss (%).

**Supplementary Table S6:** Birthweight-adjusted sensitivity analysis examining associations between feeding mode, maternal and neonatal characteristics, historical crisis exposures, and high neonatal weight loss (>10%).

**Supplementary Table S7:** Stratified logistic regression analyses of the association between breastfeeding and high neonatal weight loss across neonatal vulnerability groups, infant sex, and historical exposure strata, with Cochran–Q heterogeneity tests.

**Supplementary Table S8:** Comparison of maternal and neonatal characteristics between infants with one versus two available postnatal weight measurements.

**Supplementary Table S9:** Comparison of maternal and neonatal characteristics between infants with both postnatal weight measurements recorded exactly on days 5 and 10 and infants with other measurement patterns.

**Supplementary Table S10:** Sensitivity analyses restricted to infants with both postnatal weight measurements recorded exactly on days 5 and 10. Gestational-age-adjusted linear and logistic regression models were repeated using the same model specifications as in the primary analyses.
